# Dynamic Analyses of Contagion Risk and Module Evolution on the SSE A-Shares Market Based on Minimum Information Entropy

**DOI:** 10.3390/e23040434

**Published:** 2021-04-07

**Authors:** Muzi Chen, Yuhang Wang, Boyao Wu, Difang Huang

**Affiliations:** 1 School of Management Science and Engineering, Central University of Finance and Economics, Beijing 102206, China; zizizhuzhu0320@163.com (M.C.); dahang608@163.com (Y.W.); 2 Department of Econometrics and Business Statistics, Monash University, Melbourne 3145, Australia

**Keywords:** map equation, minimum information entropy theory, module detection, LASSO method, industry aggregation, network analysis

## Abstract

The interactive effect is significant in the Chinese stock market, exacerbating the abnormal market volatilities and risk contagion. Based on daily stock returns in the Shanghai Stock Exchange (SSE) A-shares, this paper divides the period between 2005 and 2018 into eight bull and bear market stages to investigate interactive patterns in the Chinese financial market. We employ the Least Absolute Shrinkage and Selection Operator (LASSO) method to construct the stock network, compare the heterogeneity of bull and bear markets, and further use the Map Equation method to analyse the evolution of modules in the SSE A-shares market. Empirical results show that (1) the connected effect is more significant in bear markets than bull markets and gives rise to abnormal volatilities in the stock market; (2) a system module can be found in the network during the first four stages, and the industry aggregation effect leads to module differentiation in the last four stages; (3) some stocks have leading effects on others throughout eight periods, and medium- and small-cap stocks with poor financial conditions are more likely to become risk sources, especially in bear markets. Our conclusions are beneficial to improving investment strategies and making regulatory policies.

## 1. Introduction

From June 2014 to June 2015, the Shanghai Stock Exchange (SSE) A-shares index increased by 57%. The market then experienced three large-scale collapses during the following half year from June 2015 to January 2016, where the index decreased by 49% [1]. During these events, the SSE A-shares market plummeted with high volatility and lost about 36 trillion Renminbi (RMB). Such abnormal fluctuations in the stock market were also accompanied by the highly synergistic effect of the rise and fall of the stock market, further increasing the stock market’s volatility [2].

Although the China Securities Regulatory Commission has conducted a comprehensive reform since 2005, there are repeated abnormal volatilities over the transition period between the bull and bear market in the SSE A-shares market, partly due to the immaturity of the Chinese market in terms of the traders, trading system, market system, and regulatory system [3]. During expansions (bull markets), stocks in the A-shares market display blow-out increases, accumulating bubbles, and financial risks. During recessions (bear markets), fire sale trading triggers the declines in stock liquidity and spreads the financial risk throughout the entire financial system [4]. More importantly, this highly volatile phenomenon is also prevalent in emerging stock markets, such as the dramatic plunge of the Russian market in 2018 and multiple circuit breaks in the Brazilian market between 2020 and 2021.

As the second-largest economy globally, China devotes itself to integrating into the global finance market. Specifically, a series of policy measures—including establishing the Renminbi Qualified Foreign Institutional Investor (RQFII) program, opening the Shanghai–Hong Kong Stock Connect, and continuously raising quotas in the Qualified Foreign Institutional Investor (QFII) program and the Qualified Domestic Institutional Investor (QDII) scheme—are adopted to significantly strengthen the connections between China and the rest of the world [5]. The evolution of the SSE A-shares market over good and bad periods is related to the reform of the Chinese stock market and has profound influences on the international capital and cross-border spillover risk [6,7].

Traditional econometric measurements, including the Pearson’s correlation and Granger causalities, qualify the pairwise relationship of two concerned stocks in a financial market without considering the potential influence from rest ones in the same system [8,9,10]. Although the multivariate regression model can characterise interactions between equities in a systemic way, this framework may fail to effectively fit the financial data due to the high-dimensional problem that limited observations are used to estimate a significant number of parameters reflecting relationships of stocks [11,12,13,14]. To overcome these above issues, we use the Least Absolute Shrinkage and Selection Operator (LASSO) method to model the SSE A-shares market’s network by measuring statistically significant connections in the equities system and shrinking insignificant ones into zeros [6,15]. More importantly, we also use the Map Equation method to conduct a dynamic analysis of financial contagion patterns in the SSE A-shares market. Empirical results reveal a gradually significant contagion pattern (i.e., the industry differentiation) in the system since 2014, where stocks from the same industry category tend to behave similarly. Compared with large-cap stocks, medium- and small-cap stocks react to financial risks more distinctly and function as financial contagion channels. Meanwhile, the long-term study based on spillover risks and critical nodes in contagion paths can help investors and policy makers better understand the Chinese stock market’s microstructure and the potential mechanism of the highly volatile phenomenon, which is also meaningful to other emerging financial markets and the stability of the global finance market.

## 2. Literature Review

The interactions between stocks and contagion risks are essential to understand the stock market fluctuations and global financial crisis [16,17,18]. The classical econometric methods rely on pairwise measurements including Pearson’s correlation to describe the relationships within the network system [19,20,21,22]. Naoui et al. [23] used the DCC-GARCH model to study the pairwise relationships of stock index returns of different regions over the subprime mortgage crisis and found that the United States is an essential source of contagion during this crisis. Selecting three fields—industry, banking, and public utilities—as research objects, Grout and Zalewska [24] showed that industrial market risks increase during the crisis. Bernal et al. [25] introduced the CoVaR method to measure the relationships among stock returns in the banking, insurance, and other financial sectors during the financial crisis. Das et al. [26] proposed a mixed-frequency-based regression approach, derived from functional data theories, to analyse the influence of global crises on stock market correlations between G7 countries.

Classical econometric approaches mainly focus on the direct relationship between two financial agents but fail to reflect potential influences from the complex system on the direct connection [27,28,29,30]. However, such underlying interactions can be well revealed under the networking framework by investigating financial networks’ topological properties and statistical characteristics. Liu and Tse [31] used five years of stock index data from 67 countries and use Pearson’s correlation to generate a complex network. Gong et al. [32] employed the transfer entropy method to analyse interactions between national stock markets and discovered that countries affected by the crisis become closer to each other and the total network connectedness rises during the crisis. Chen et al. [33] used complex network theories to measure systemic risks in the stock market and developed dynamic topological indicators to analyse financial contagion and qualify the magnitude of systemic risks. Coquidé et al. [34] analysed the world trade network’s risk contagion during the global crisis and explored the structural trading dependencies between countries. Constructing undirected and directed volatility networks of the global stock market, Lee et al. [35] applied machine learning methods to study network indicators for establishing an international financial portfolio management approach. Liu et al. [36] investigated 6600 banks’ decision rules and behaviours reflected in quarterly balance sheets to construct interbank networks and further examine how financial shocks spread through financial contagion. Kumar and Deo [37] applied random matrix theories to study the topological properties of a network consisting of 20 nations and analysed communities in the generated network under different thresholds. Li et al. [38] discussed the relationships between listed energy companies and their shareholders under the networking framework. Empirical studies show that most energy investment is concentrated in a few countries, and some islands or regions play irreplaceable roles in tax avoidance. Paltalidis et al. [39] employed the maximum entropy method to study the systemic risk and analyse the vulnerability of the Euro area’s financial network. Based on the United Nations COMTRADE database, Coquidé et al. [40] used the simplified Google matrix (REGOMAX) algorithm to analyse the multiproduct world trade network from 2004 to 2016 and provided a more detailed analysis of trade influence propagation.

The structural differences between bull and bear markets have been widely discussed. Zhang et al. [41] used VaR and CoVaR models to build a new investor sentiment index and applied it to predict stock prices and estimate systematic risks in bull and bear markets. Wen et al. [42] adopted Pearson’s correlation and the copula model to investigate the structural characteristics of equity markets in Europe, Asia, and Africa. Empirical results suggest that European markets are more influential than Asian and African markets during booming and recession periods. Given the rapidly growing weight of the Chinese economy globally, many researchers focus on the risk transmission mechanism during stock market crashes. By constructing coattention networks for the Chinese stock market, Chen et al. [43] discovered the structural differences of bull and bear markets and utilised such differences to predict stock returns. Dividing the year 2015 into four periods (the tranquil, bull, crash, and postcrash), Wang and Hui [44] used kernel estimation to build the information transfer network for studying information transition before and after the 2015 crash. Adopting the mutual information and symbolisation methods, Khoojine and Han [45] generated minimum spanning trees of the top 110 companies listed on the China Securities Index 300 from January 2014 to December 2017 to study the differences in topological characteristics of preturbulence, turbulence, and post-turbulence networks.

Using networking methodologies to study stock markets may suffer from the high-dimensional problem that traditional estimations are not consistent. The number of stocks (*N*) in a market is comparable to that of observations (*T*) over a specific period (e.g., the bull and bear market), hence, the size of unknown parameters ($O(N^{2})$) is comparable to that of data (O(NT)). The LASSO method provides a promising solution to alleviate the high-dimension problem when building financial networks. Xu et al. [46] utilised the LASSO–CoVaR model to construct a financial network for the Chinese stock market between 2010 and 2017 and analysed financial institutions’ status and role in crises. Using the data on the subprime mortgage crisis, Demirer et al. [47] adopted the LASSO–VAR method to analyse the global system’s static and dynamic connectedness.

Applying the LASSO method, we generate the stock networks and community structures to analyse financial systems’ evolution. We further introduce the Map Equation method to study the SSE A-shares market’s dynamic changes and its differentiation in industries. The Map Equation method is based on information theories and has been further improved in subsequent studies [48,49,50], it is widely used in biological, information, and social networks [51]. This method utilises the probability flow of random walks on a network as a proxy for information flows in the entire system to decompose the network into different modules by compressing the probability flow description. The Map Equation approach is also adopted to investigate risk transmission in financial settings, which makes it possible to analyse the overnight market risk path of commercial banks [52], the centrality of financial network institutions and measurements on systemic risks [53], and the financial integration of banks in developed regions before the subprime crisis [54].

## 3. Methodologies

### 3.1. LASSO Estimation and Network Construction

Traditional econometric methods use pairwise measurements, such as Pearson’s correlation and Granger causalities, to qualify interactions between stocks. However, these approaches only measure the direct relationship between the two stocks without considering the potential influence from rest stocks through a systematic perspective. When using Pearson’s correlation, the correlation between stock A and B may indirectly derive from stock C, which is highly correlated with stock A and B separately. Granger causalities are also inappropriate for describing sophisticated linkages in financial markets due to theoretical reasons. For any given stock pair, the white noise assumption in the Granger causality test implies that there are no connections between the concerned two stocks and the rest. In other words, these two stocks are presumed to be isolated from the entire system, and this may contradict the networking structure in financial markets. Although classic multivariate regression models can overcome the above two drawbacks, they fail to fit data in the high-dimensional situation that the number of stocks (*N*) is proportional to that of observations (*T*) (i.e., N=O(T)). As a promising solution, the LASSO method chooses an absolute value function as the penalty term to screen significant variables and shrinks insignificant ones into zeros, which can solve the fitting problem in high-dimensional cases.

We consider a multivariate linear regression model to reveal relationships in the stock market from a systemic way. For stock *i*, the model is

(1) $$r_{it}=r_{1t}\beta_{i1}+r_{2t}\beta_{i2}+\cdots +r_{i-1,t}\beta_{i,i-1}+r_{i+1,t}\beta_{i,i+1}+\cdots +r_{Nt}\beta_{iN}+\varepsilon_{it},$$

where $r_{it}=\ln P_{it}-\ln P_{i,t-1}$ is the log return of stock *i*, $P_{it}$ is the stock price of *i* at time *t*, *N* is the number of stocks, and $\varepsilon_{it}$ is the error term. Since unknown parameters $\beta_{-i}=\left\{\beta_{ij},j=1,\cdots ,i-1,i+1,\cdots ,N\right\}$ qualify the stock relationships in the financial market, the LASSO method is adopted to estimate those statistically significant parameters and shrink those insignificant ones into zeros. The LASSO estimate is

(2) $${\hat{\beta }}_{-i}=\underset{\beta_{-i}}{\mathrm{argmin}}\left\{\frac{1}{2T}\sum_{t=1}^{T}{\left(r_{it}-\sum_{j\neq i}r_{jt}\beta_{ij}\right)}^{2}+\lambda \sum_{j\neq i}|\beta_{ij}|\right\},$$

where *T* is the number of observations and λ is the tuning parameter preset by the cross-validation method (This paper uses the glmnet package in R to obtain LASSO estimations). We present the estimation of $\beta_{-i}$ in Appendix B. The above procedure was repeated for all stocks. Then, the adjacency matrix $A={\left\{a_{ij}\right\}}_{N\times N}$ for the financial market is defined as

(3) $$a_{ij}=\left\{\begin{array}{cc} 1, & \mathrm{if}\;{\hat{\beta }}_{ji}\neq 0;\\ 0, & \mathrm{if}\;{\hat{\beta }}_{ji}=0. \end{array}\right.$$

Equation (3) suggests that a direct link from stock *i* to *j* exists if and only if its corresponding LASSO estimate ${\hat{\beta }}_{ji}$ is nonzero.

### 3.2. Module Detection Based on the Information Entropy Methods

This paper introduces the Map Equation method to detect modules in the SSE A-marker network over different periods and further explores the evolution of modules. The Map Equation algorithm (InfoMap algorithm), initially proposed by Rosvall and Bergstrom [48], is based on a formula to evaluate the effectiveness of a module structure in describing the path of a random walker around the network. The random walk is used to simulate information (risk) transmission in the entire system (the stock market). Based on Huffman coding, the Map Equation algorithm adopts a two-level code to describe the random walk path: the high-level codes distinguish modules in the network (i.e., index codes) and the low-level codes represent node names that are unique in the same group (i.e., module codes). The Map Equation algorithm aims to discover an information (risk) map that gets rid of unnecessary details by minimising the amount of information needed to describe the random walk path and highlights critical modules where nodes in the same group develop stronger interior relationships than outside nodes.

Since the real relationships between stocks are not observable, we first examine the discovered network structure in Equation (3) to simulate the real one and calculate the visit frequencies of a random walker travelling in the system. Similar to the method used in [54], we convert the adjacency matrix A defined in Equation (3) to the Markov transition probability matrix Π to depict the random walk path. Since A may be nonsymmetric, we consider the follow systemic matrix:

(4) $$\begin{array}{c} V=\left\{v_{ij}\right\}=\left[\begin{array}{cc} 0_{N} & A\\ A^{⊤} & 0_{N} \end{array}\right]\in R^{2N\times 2N}, \end{array}$$

where $0_{N}\in R^{N\times N}$ is a matrix with zeros. Then, the Markov transition probability matrix is defined as

(5) $$\begin{array}{c} \Pi ={\left[\pi_{ij}\right]}_{2N\times 2N}={\left[\frac{v_{ij}}{\sum_{k=1}^{2N}v_{kj}}\right]}_{2N\times 2N}. \end{array}$$

Let $p_{i}$ be the visit frequency of a random walker to the node *i*. Mathematically, we can calculate $p_{i}$ by considering the dominant eigenvector of the Markov transition probability matrix,

(6) $$\begin{array}{c} P=\Pi P, \end{array}$$

where $P={\left(p_{1},\cdots ,p_{2N}\right)}^{⊤}$.

Given the visit frequencies P and a module structure M with *m* modules, the exiting frequency of the traveller from module α is given by

(7) $$q_{\alpha ↷}=\sum_{i\in module\;\alpha }\sum_{j\notin module\;\alpha }\pi_{ij}p_{i},$$

and the exit frequency of the travel from *any* module is given by

(8) $$\begin{array}{c} q_{↷}=\sum_{\alpha =1}^{m}q_{i↷}. \end{array}$$

Moreover, the frequency at which the random walker uses module α’s codes is given by

(9) $$p_{⥀}^{\alpha }=q_{\alpha ↷}+\sum_{i\in module\;\alpha }p_{i}.$$

The probabilities $p_{⥀}^{\alpha }$ and $q_{\alpha ↷}$ measure the frequency of using module codes. Next, we need to know the costs to access these codes. According to Shannon’s coding theorem, for a random variable *z* having *n* states with probabilities $p_{k}$, the average length of the code word cannot be less than the entropy of *z*, defined by

(10) $$H\left(z\right)=-\sum_{k=1}^{n}p_{k}\log\left(p_{k}\right).$$

Then, the minimum average length for index codes and module codes are given by

(11) $$H\left(Q\right)=-\sum_{\alpha =1}^{m}\frac{q_{\alpha ↷}}{q_{↷}}\log\left(\frac{q_{\alpha ↷}}{q_{↷}}\right)$$

and

(12) $$H\left(P^{\alpha }\right)=-\frac{q_{\alpha ↷}}{p_{⥀}}\log\left(\frac{q_{\alpha ↷}}{p_{⥀}}\right)-\sum_{i\in module\;\alpha }\frac{p_{i}}{p_{⥀}^{\alpha }}\log\left(\frac{p_{i}}{p_{⥀}^{\alpha }}\right),$$

where H(·) is the entropy function defined in Equation (10) and H(Q) and $H(P^{\alpha })$ reflect the encoding effectiveness of the entire module structure *M* and the specific module α, respectively.

Consequently, the minimum average length of the random-walk path under the given module structure *M* is given by

(13) $$L\left(M\right)=q_{↷}H\left(Q\right)+\sum_{\alpha =1}^{m}p_{⥀}^{\alpha }H\left(P^{\alpha }\right).$$

Here, L(M) is the weighted sum of two information entropy parts. One is the average code length of the module name (index codes) and the other one is the average code length of node names in different modules (module codes). The weights are proportions of average code lengths of module and node names. When minimising the L(M), the network achieves the minimum entropy and the corresponding module division is stable. The map equation algorithm uses these criteria to compare different module divisions in practice and select the optimal one.

### 3.3. Network Topological Indicators

Based on the detected module structures, we further investigate the topological properties of generated networks to study the contagion effect in the SSE A-shares market.

**Average** **shortest** **path** **length**$L=\frac{2}{N(N-1)}\sum_{j\neq i}d_{ij}$, where *i* and *j* are two stocks (nodes) in the SSE A network and $d_{ij}$ is the shortest path between nodes *i* and *j*. A smaller length means faster information or risk transmission in the network.
**Clustering** **coefficient**$C_{i}=\frac{2n_{i}}{k_{i}\left(k_{i}-1\right)}$, where $k_{i}$ is the number of nodes directly connecting to node *i* and $n_{i}$ is the number of edges between $k_{i}$ neighbours of node *i*. A higher value implies better network connectivity.
**Network** **diameter**$\mathrm{Diameter}=\max_{1\leq i,j\leq N}d_{ij}$. A smaller value implies faster information or risk transmission speed.
**Network** **density**$\mathrm{Density}=\frac{\sum_{i,j}a_{ij}}{N(N-1)}$, where $a_{ij}$ is defined in Equation (3). A higher density implies a closer relationships between nodes.
**Relative** **degree** **centrality**$C_{RD}\left(i\right)=\frac{k_{i}}{N-1}$. The high relative degree centrality implies an important influence from the corresponding node on the network.
**Relative** **betweenness** **centrality**$C_{RB}\left(i\right)=\frac{2}{\left(N-1)(N-2\right)}\sum_{j<k}\frac{g_{jk}\left(i\right)}{g_{jk}}$, where $g_{jk}\left(i\right)$ is the number of shortest paths connecting nodes *j* and *k* and passing through node *i*. This indicator measures the “bridge” role of node *i* in the network.
**Relative** **closeness** **centrality**$C_{RC}\left(i\right)=\frac{N-1}{\sum_{j=1}^{N}d_{ij}}$ that measures how close node *i* is to all other nodes in the network. The high value of relative closeness centrality implies close connections between node *i* and other nodes.
**Degree** **centralisation>**$C_{D}=\frac{\sum_{i=1}^{N}\left(C_{RD}\left(\max\right)-C_{RD}\left(i\right)\right)}{\max\left[\sum_{i=1}^{N}\left(C_{RD}\left(\max\right)-C_{RD}\left(i\right)\right)\right]}$, where the numerator is the sum of differences between the maximum degree centrality $C_{RD}\left(\max\right)=\max_{1\leq i\leq N}C_{RD}\left(i\right)$ and the degree centrality of each node $C_{RD}\left(i\right)$, and the denominator is the maximum value of the numerator in theories. This indicator describes the centrality of the whole network.
**Betweenness** **centralisation**$C_{B}=\frac{1}{N-1}\sum_{i=1}^{N}\left(C_{RB}\left(\max\right)-C_{RB}\left(i\right)\right)$, where the numerator theoretically represents the sum of the difference between the maximum intermediate centrality and the intermediate centrality of each node, and the denominator represents the maximum of the sum of the differences. This indicator describes the degree to which the network relies excessively on a node to transfer relations.
**Closeness** **centralisation**$C_{C}=\frac{2(N-3)}{\left(N-1)(N-2\right)}\sum_{i=1}^{N}\left(C_{RC}\left(\max\right)-C_{RC}\left(i\right)\right)$, which describes the centralised trend in the network.

## 4. Empirical Results

### 4.1. Stock Market Data

This paper uses the SSE A-shares market’s weekly closing prices from 2005 to 2018 and refers to important financial events and policies to divide the entire period into eight stages according to bull and bear markets.

As shown in Table 1, stages 1 and 5 are typical rapidly rising bull markets and stages 2 and 6 are steeply declining bear markets. By contrast, stages 3 and 7 witness moderate increases in the market, and the market experiences a fluctuating decline in stages 4 and 8. Overall, the surge and plummet can be found in the bull market (stages 1 and 5) and the bear market (stages 2 and 6), respectively, whereas the stock market consistently fluctuates in stages 3, 4, 7, and 8. Based on the above division, we aim to utilise information entropy to discover the evolution of modules in the SSE A-shares market in expansions and recessions.

The SSE A-shares backward closing prices from 2005 to 2018 are downloaded from the wind database. Given the long period of research data, some stocks are out of the discussion in this paper because of the following reasons.

1. Missing values. Until 18 December 2019, 1547 stocks were traded on the SSE A-shares market. Due to the late listing and suspension of trading, some of these stocks were removed in advance to compare stock market networks in different stages.
2. Stocks prefixed with “ST” or “*ST”. Designated as Special Treatment (ST) by the stock exchanges for warning investors, these stocks face delisting risks and show distinct patterns from normal stocks.
3. Stocks whose returns maintain at zeros over a long period for the long-term suspension or other reasons were excluded in this paper to prevent misleading information.

As a result, 716 stocks were selected to construct the SSE A-shares network. For the stock *i*, its log return is calculated by

(14) $$r_{it}=\ln P_{it}-\ln P_{i,t-1},$$

where $P_{it}$ is the price of stock *i* at time *t*.

### 4.2. Module Analysis of the SSE A-Shares Network Based on the Minimum Entropy

**Figure 1:**
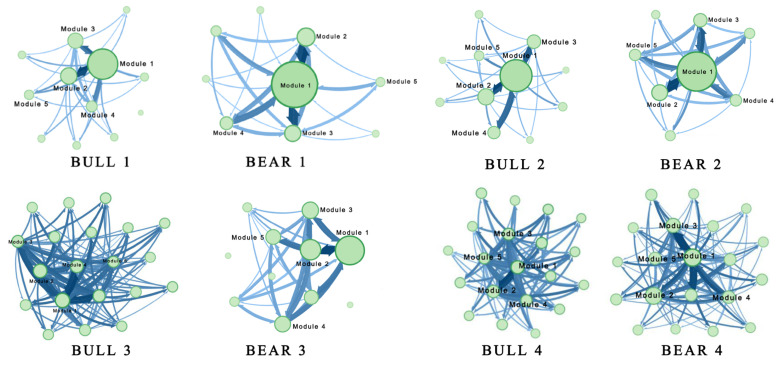
Module divisions in eight periods based on the Map Equation algorithm. Nodes represent modules, directed links indicate directions of the information flow, and the thickness of links demonstrates the transmission probability of risk between different modules. The node size is proportional to the information flow in a module that includes the information flow out of and within the module. Specifically, the node’s boundary thickness is proportional to the information flow out of the module, corresponding to the probability that risks transmit to other modules. The interior area of a node is proportional to the module’s information flow, corresponding to the probability that risks stay in the module.

**Figure A1:**
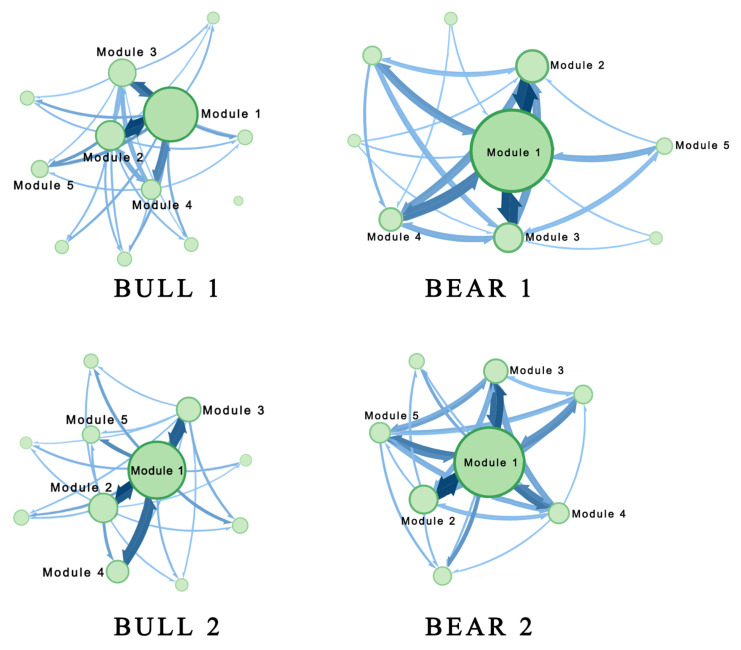
Module divisions in eight periods based on the Map Equation algorithm. Nodes represent modules, directed links indicate directions of the information flow, and the thicknesses of links demonstrate the transmission probability of risk between different modules. The node size is proportional to the information flow in a module that includes the information flow out of and within the module. Specifically, the boundary thickness of a node is proportional to the information flow out of the module, corresponding to the probability of risk of transmission to other modules. The interior area of a node is proportional to the information flow within the module, corresponding to the probability that risks stay in the module.

**Figure A2:**
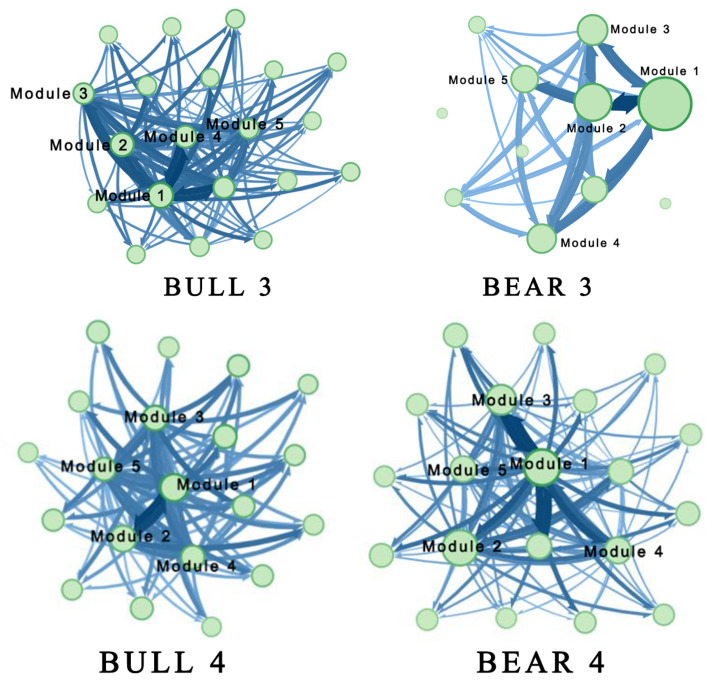
Module divisions in eight periods based on the Map Equation algorithm. Nodes represent modules, directed links indicate directions of the information flow, and the thicknesses of links demonstrate the transmission probability of risk between different modules. The node size is proportional to the information flow in a module that includes the information flow out of and within the module. Specifically, the boundary thickness of a node is proportional to the information flow out of the module, corresponding to the probability of risk of transmission to other modules. The interior area of a node is proportional to the information flow within the module, corresponding to the probability that risks stay in the module.

To analyse the characteristics of modules in the SSE A-shares market, we employed the Map Equation method to detect modules in the financial system in different periods and present visualisation results in Figure 1 (Figure A1 and Figure A2 in Appendix A provide the full-size version of Figure 1). Each module is represented by a node whose area is proportional to its information flow, reflecting its status in the network. The information flow of a module includes two parts: the information flow out of the module, which is proportional to its boundary thickness and can represent the probability of risk passing from the module to other modules; and the information flow staying in the module, which is proportional to the area of the inner circle area that can indicate the probability that the risk remains in the module. The connection thickness among modules suggests the probability of risk transmission in different sectors. The thicker the connection, the greater the contagion probability. Besides, the arrow indicates the direction of the risk of contagion.

As shown in Figure 1, the module numbers in bear markets are generally smaller than those in bull markets, and more stocks belong to the same module in bear periods, indicating closer interior connections and higher internal contagion in modules. The number of modules in the stock market gradually increases, implying that the continuous development of the capital market leads to differentiation in the patterns of stock returns and the risk of contagion.

To investigate how modules develop and evolve over the eight periods, we consider stock categories in different modules and present results of Bear 1, Bull 4, and Bear 4 in Table 2, Table 3 and Table 4, respectively.

As shown in Table 2, the SSE A-shares network in Bear 1 can be divided into nine modules, and three modules are center out of the discussion as they only include one stock. M1 has the largest size (608 stocks) in the network, and includes and captures 85.87% of the network’s information. Conversely, the remaining modules contain less information, and the differentiation effect caused by industry aggregation is not significant. More importantly, the industry distribution of M1 in Bear 1 is analogous to that of the entire SSE A-shares market distribution. Consequently, M1 can be viewed as a system module, and other stocks that are not in the first module are peripheral in the stock market. Similar patterns can also be found in the other first four stages.

Unlike the first four stages, the last four stages except for Bear 3 present the significant industry differentiation of the modules, as shown in Table 3 and Table 4.

Table 3 suggests the nine largest modules in the Bull 4 stage network, and the remaining 42 modules are out of discussion due to involving few stocks. The differentiation of the modules displays a significant industry aggregation phenomenon. Health care is the dominant industry category in M1 and is key to the information flow and spillover risk. Given by Figure 1, the closed connections between M1 and M2 imply the high potential risk of contagion between the health care and consumer discretionary industries because of the distinct overlap of these two industries in the industry chain.

The Bear 4 stage can be divided into 46 modules, and the top nine modules with the largest sizes capture 57.44% of the network information. The industry categories of stocks in these nine modules are provided in Table 4. M1 mainly consists of stocks from materials, industry, and consumer staples. The dominant industries in M2, M3, M4, M5, and M6 are consumer discretionary and consumer staples, information technology and industrials, industrials, consumer staples, and materials.

Table 3 and Table 4 demonstrate the significant industry aggregation effect in the Bull 4 and Bear 4 period. With the development of the stock market, the industry aggregation results in module differentiation in SSE A-shares because stocks from the same industry category share similar macro fundamentals.

Based on the Map Equation method and minimum information entropy, we discuss the module divisions in eight periods and further analyse the risk spillover among modules.

Table 5 compares the top five modules with the highest proportion of information in bull and bear periods. The first column of Table 5 shows the modules ranked by information flow from high to low, the second column represents the number of stocks (nodes) involved in the module, and the third column represents the number of information transmission linkages (links) within the module. Table 5 suggests that significant structural differences exist between the first four stages and the latter four stages. In the first four stages, the proportions of stock and link numbers within the largest module account for more than 70% of the entire SSE A-shares network, whereas the proportions in other modules are low. In other words, the SSE A-shares market does not show significant differentiation in the first four stages: the largest module, having the highest information flow, largely represents the entire network, and more than 70% of the stocks belong to this module. However, the SSE A-shares market experiences significant structural differentiation in the last four stages. Specifically, the largest five modules in Bull 3, Bull 4, and Bear 4 share comparable sizes in node and link numbers. Compared with the other three stages, the Bear 3 period is closer to the first four stages because the rapid deleveraging effect in this stage leads to abnormal fluctuations and increases the system’s connected effect.

We use the information flow to illustrate the spillover effect of risks within and between modules in Table 6: the first column represents the modules ranked by information flow from high to low; the second column represents the proportions of information within modules to the total information; the third (fourth) column represents the proportion of information flowing into (out of) each module. Table 6 presents similar results to Table 5. In the first four stages, the information in the largest module accounts for at least 70% of the entire market, indicating that most stocks can be grouped and insignificant differentiation exists in the stock market. Conversely, in the last four stages, except for the Bear 3 stage, the most extensive module merely contains approximately 10% information, suggesting significant differentiation in the stock market. Such differentiation derives from industry agglomeration in that stocks from the same industry are more likely to form modules (See details in Table 3 and Table 4). Moreover, Bear 3 can be viewed as a transition period between the first and last four stages since the information proportion of M1 is about 50%. During this period, given the excessive accumulation of preleverage and the speed of the deleveraging process, abnormal fluctuations occurred in the stock market, and the strengthened connected effect in the system further increases the abnormal volatility of the stock market, resulting in a more significant loss in the financial system.

In summary, more than 70% of stocks in the SSE A-shares market belong to the same module in the first four periods, which can be viewed as the system module. Stocks in the system module are less connected with others, and those outside the system module are pericardial in the network, implying the relatively low contagion risk and weak connections. As China’s financial market develops, the system module is gradually differentiated into several small parts based on industry categories. Stocks’ from the same or related industries grouping in modules make the industry aggregation phenomenon more significant. In an extreme situation like the Bear 3 stage, abnormal fluctuations and large-scale declines in the market lead to the formation of a small system module whose higher status accelerates risk contagion.

### 4.3. Topological Properties of SSE A-Shares Networks

We utilise topological indicators in Section 3.3 to investigate the SSE A-shares market’s characteristics over bull and bear markets. Details are presented in Table 7 and Table 8.

As shown in Table 7 and Table 8, network densities of bear markets are higher than those of bull markets, and network diameters of bear markets are lower than those of bull markets, suggesting that stocks have stronger connections with others in the bear market. Affected by the subprime mortgage crisis and the deleveraging of capital allocation, the SSE A-shares market experiences large-scale collapses and shows a significant connected effect. Meanwhile, investors are more sensitive to market information in these two periods, and hence, tend to adopt similar strategies to avoid risks.

In the entire period, the lengths of the average shortest path in the SSE A-shares networks are between two and three, meaning that approximately two or three intermediate stocks can connect any stock pairs. The clustering coefficients of bear markets are generally higher than those of bull markets, reflecting the more distinct connected effect in recessions. Bear 1 was during the subprime mortgage crisis, Bear 1 was affected by the European debt crisis, and Bear 3 experienced the “thousand-share limit-down" after the stock market deleverages in 2015, which reflects that a financial crisis enhances the small-world effect of the SSE A-shares network.

Regarding the difference between bull and bear markets, the average degrees of stocks in bear markets are greater than those in bull markets. In the bear market, the core stocks have more leading influence, and more synchronous changes appear in the market. Further, closeness centralities are relatively high, which means that the reachable distances of risk propagation are relatively short and risks can transmit to most stocks from the source via a short distance.

From the structural differences between bear markets, Bear 1 and Bear 3 show rapid declines with large volatility, while Bear 1 and Bear 4 fall with fluctuations and have small volatility. Therefore, structural differences exist between these two types of bear markets. The low out-degree centralisations of Bear 1 and Bear 3 reflect the marginal differences between the out-degree centrality of each node and the maximum out-degree centrality. Therefore, most stocks in these two periods have relatively high out-degrees, and risks are more likely to transmit to other stocks through these stronger connections, accelerating the propagation of risks. By contrast, the Bear 1 and Bear 4 markets show different patterns. The low in-degree centralisation and high out-degree centralisations reflect that most stocks have relatively high in-degrees but low out-degrees, suggesting that stocks in the network absorb risks and prevent the spread of risks. Bear 1 and Bear 3 are periods with abnormal fluctuations due to the impact of the subprime mortgage crisis and rapid deleveraging in 2015. In Bear 1 and Bear 1, it is hard to identify critical stocks leading to the massive collapse, making the supervision of risks more challenging. Conversely, the less-connected structure of the SSE A-shares networks in Bear 1 and Bear 4 lowers the transmission risks and benefits of identifying the risk source and restraining the network’s spread of risk.

### 4.4. Analysis of Core Stocks in Bull and Bear Markets

We use three types of centralities to measure stocks’ influence in the SSE A-shares market to identify core stocks in the network and investigate how these stocks transmit risks over different periods. Table 9, Table 10 and Table 11 list stocks with the top five relative degrees, betweenness, and closeness centralities in eight stages, respectively, where the first column reports stock codes in the SSE A-share market and the second column gives the corresponding industry categories. Stocks in these tables are referred to as core stocks due to their distinct influence in specific periods.

Table 9, Table 10, Table 11, Table 12 and Table 13 present the top five stock lists in terms of the relative degree centrality, relative betweenness centrality, relative closeness centrality, PageRank, and CheiRank. First, mutual components of industry categories in Table 9, Table 10 and Table 11 demonstrate that high-influence stocks are mainly from four industry categories: consumer discretionary, health care, materials, and industrials, corresponding to the system module and the industry aggregation phenomenon demonstrated in Table 2, Table 3 and Table 4. Second, the overlap proportions of three stock lists measured by different centralities are relatively high in the same period, about 90% of stocks in degree and closeness centralities lists are the same. Compared with degree centralities and closeness centralities measuring direct connections and transmission distances, betweenness centralities reflect the importance of stocks in contagion paths. Therefore, distinct aspects of the three types of centralities decrease the three stock lists’ overlap proportions. It is worth noting that a few stocks simultaneously appear in three centrality lists in the same stage, which implies that these stocks play irreplaceable roles in the financial system and are the main driving factors of significantly increasing the network connectivity. Third, although bull and bear markets share noticeably different core stocks, about 22.5% of stocks (e.g., codes 600861 and 600356) are ranked in the top 20 centrality lists over the entire period, playing unique roles in leading the SSE A-shares market. Lastly, PageRank reflects how likely a given stock is influenced by other stocks, while CheiRank measures the impact of a given stock on the rest ones (We adopted methods from Coquidé et al. [34,40] to compute the PageRank and CheiRank). Table 12 shows different results from other measurements and lists those vulnerable stocks in eight stages. By contrast, Table 13 shares a similar list with Table 9, suggesting that core stocks’ high connectivity mainly derives from their profound influence on the rest of stocks.

We present the averages of financial indicators of stocks with the top 20 centralities in different stages in Table 14 to further investigate the characteristics of core stocks. For comparisons, Table 14 also provides the financial indicator averages of all SSE A-shares in parentheses.

Table 14 suggests that 67% of the top 20 stocks in the core nodes from different perspectives belong to small and mid-cap stocks rather than large-cap stocks, and further analysing their financial status through Table 14 shows that although high market capitalisation stocks such as PetroChina and ICBC have a greater influence on the index due to the index weighting design, their correlation with other stocks is not strong. Further summarising the financial characteristics of the core nodes, in terms of solvency, the average short-term solvency of the core nodes is weak. The current ratio and a quick ratio of two-thirds of the core nodes are lower than the average of all SSE A-share stocks in the same period, with nearly one-third of the core nodes having a current ratio below 1; in terms of profitability, the return on net assets of the core nodes declines year by year and is much lower than the average of SSE A-shares in the same period. In terms of profitability, the ROE of two-thirds of the core nodes is lower than 10% and the profitability is lower. On the contrary, many large-cap stocks, although they have a greater role in guiding the stock index, have less influence on the system-wide resonance and linkage effect, which is consistent with the frequent occurrence of the Chinese stock market. This is in line with the “two-eight divergence” that often occurs in the Chinese stock market, i.e., when the heavyweight stocks show significant gains, small- and mid-cap stocks tend to be less volatile or even decline. This phenomenon tends to be different from the performance of financial markets in developed countries, and Bosma et al. [55] found through a correlation network among as many as 186 financial institutions in the 2007–2008 U.S. financial crisis that systemically important financial institutions with large sizes and market capitalisations are also at a more central position in terms of the correlation status of their stock returns. Overall, there tends to be a higher overlap between “too big to fail” and “too correlated to fail” institutions and stocks in developed country stock markets, i.e., stocks with larger market capitalisations tend to be at the centre of correlation and connectivity in stock markets.

The main reason for this discrepancy is that, as a representative of the emerging stock market, the composition of investors in Chinese A-shares differs significantly from that of developed economies. The share of individual investors is more than 80%, which, together with the immaturity of the regulator, has contributed to the speculative atmosphere in the market. On the one hand, when the market rises, shareholders follow the trend, and these stocks are particularly favoured by ordinary investors because of their relatively small capitalisation, which makes them vulnerable to capital control and higher increases in the short term, thus further boosting share prices; on the other hand, however, because such share price increases do not come from the company’s own performance improvement, it is difficult to receive long-term investors’ attention, and only blind increases will occur only. As this kind of stock price increase does not originate from the company’s own performance improvement, it is difficult to receive long-term investors’ attention, and the blind rise will only accumulate bubbles rapidly in the short term, laying a hidden danger for the outbreak of financial risks. After speculators sell at high stock prices, it will trigger an avalanche of ordinary stockholders to withdraw, eventually leading to a precipitous decline in stock prices—coupled with the immaturity of China’s capital market itself, market sentiment is susceptible to influence, investors are prone to overreaction, and the risk of individual stocks quickly spread throughout the market, leading to a sharp rise and fall in the entire market phenomenon.

The abnormal stock market volatility in 2015 was the superimposed effect of such highly leveraged funding and the financial market’s herding effect. In the first half of 2015, driven by multiple favourable factors such as the central bank’s interest rate cut, lowering of quotas, and speeding up of IPO approval, the stock market rose and surged under the boost of financing, financing business, and over-the-counter funding. The network structure of the first half of the year not only increased the financial risk but also made the stock market more tightly structured; with the increase in the proportion of financing leverage, the risk of blowout increased simultaneously, after which the liquidity turned from high to low due to the rapid strengthening of financial supervision in the short term and the malicious shorting by some investors in spite of the market risk, the systemic risk increased steeply, the stock price bubble was punctured, and there were many times when a thousand shares fell, which further intensified the forced closure of positions in the field financing and over-the-counter matching. This caused the stock price to cycle down, which eventually led to a dramatic fluctuation of the stock index falling by almost half within six months. With the reform and continuous improvement of China’s financial market, the market capitalisation of individual investors on the SSE dropped from 63% to 55% between 2015 and 2018, so the microstructure of the market has been differentiated in various aspects such as sectors, but overall, the institutionalisation of China’s A-share market needs to be improved in terms of both market capitalisation and trading volume.

## 5. Discussion

Our paper investigates the rise and fall of China’s stock market and explores the characteristics of this phenomenon that not only recurred during the subprime mortgage crisis in 2015 but also occurred in 2018 and 2021. As one of the most important emerging markets, the stock market in China plays a vital role in the global financial markets. Therefore, our paper also has universal significance and research value in the worldwide market. Our paper has made significant improvements in delineating the economic relevance of stocks. Using the LASSO model, economic relevance can be described from the entire stock system. The traditional measurement, such as Granger causality and Pearson relevance, is based on the pairwise relationship. Our paper is more objective and reasonable in terms of economics methodology and more in line with the accurate fitting of the measurement method. At the same time, we lay a reliable foundation for the division of research modules based on the entropy method to describe the stock correlation and to explain the important improvement and significance of the LASSO method compared with traditional methods. In the module division study based on the entropy method, we analyse the risk spillovers within and between modules, combine the entropy method to apply to the Chinese financial market, and carry out a financial analysis of the corresponding module division. We explain the correlation between these modules and the phenomenon of industry rotation. We also explore industry rotation characteristics that SMEs may establish a guiding relationship with more stocks and explain the financial indicators of this type of SME. We further show that, based on the division of social structure by entropy, China has better excavated the financial meaning behind the community’s structure.

Our results highlight that the system module is gradually differentiated into several small parts based on 249 industry categories as China’s financial market develops. There is no industry differentiation in the first four stages because of investors’ immaturity and the relative lack of depth in industry analysis. Industry differentiation emerges in the subsequent multiple stages because stocks from various industries have different degrees of consistency. As stock information is mined, investors can better identify stocks supported by policies, so significant differences emerge in stocks’ aggregation between different industries. Stocks in the same sector show a higher degree of correlation. During the risk transfer of industries, related upstream and downstream industries also show closer correlation with each other, as evidenced by the latter four stages in modules 1 and 2. The risk transmission between them can be shown, which further proves the driving effect of stock information mining on industry module differentiation. In the 2015 stock market crash, as there were several thousand stock drops, the market’s industry differentiation effect weakened and systemic risk increased again, more similar to the first four modules. The market showed a more substantial investor herding effect and irrational selling.

Our results also shed new insights on the stock markets. As is the case in China, 67% of the top 20 stocks in the core nodes from different perspectives belong to small- and mid-cap stocks rather than large-cap stocks, although high market capitalisation stocks such as PetroChina and ICBC have a greater influence on the index due to the index weighting design, their correlation with other stocks is not strong. Further summarising the financial characteristics of the core nodes, in terms of solvency, the average short-term solvency of the core nodes is weak. The current ratio and a quick ratio of two-thirds of the core nodes are lower than the average of all SSE A-share stocks in the same period, with nearly one-third of the core nodes having a current ratio below 1; in terms of profitability, the return on net assets of the core nodes declines year by year and is much lower than the average of SSE A-shares in the same period. In terms of profitability, the ROE of two-thirds of the core nodes is lower than 10% and the profitability is lower. On the contrary, many large-cap stocks, although they have a greater role in guiding the stock index, have less influence on the system-wide resonance and linkage effect, which is consistent with the frequent occurrence of the Chinese stock market. This is in line with the “two-eight divergence” that often occurs in the Chinese stock market, i.e., when the heavyweight stocks show significant gains, small- and mid-cap stocks tend to be less volatile or even decline. This phenomenon tends to be different from the performance of financial markets in developed countries, and Bosma et al. (2019) finds through a correlation network among as many as 186 financial institutions in the 2007–2008 U.S. financial crisis that systemically important financial institutions with large sizes and market capitalisations are also at a more central position in terms of the correlation status of their stock returns. Overall, there tends to be a higher overlap between “too big to fail” and “too correlated to fail” institutions and stocks in developed country stock markets, i.e., stocks with larger market capitalisations tend to be at the centre of correlation and connectivity in stock markets.

The main reason for this discrepancy is that, as a representative of the emerging stock market, the composition of investors in Chinese A-shares differs significantly from that of developed economies. The share of individual investors is more than 80%, which, together with the immaturity of the regulator, has contributed to the speculative atmosphere in the market. On the one hand, when the market rises, shareholders follow the trend, and these stocks are particularly favoured by ordinary investors because of their relatively small capitalisation, which makes them vulnerable to capital control and higher increases in the short term, thus further boosting share prices; on the other hand, however, because such share price increases do not come from the company’s own performance improvement, it is difficult to receive long-term investors’ attention, and blind increases will occur only. As this kind of stock price increase does not originate from the company’s own performance improvement, it is difficult to receive long-term investors’ attention, and the blind rise will only accumulate bubbles rapidly in the short term, laying the hidden danger for the outbreak of financial risks. After speculators sell at high stock prices, it will trigger an avalanche of ordinary stockholders to withdraw, eventually leading to a precipitous decline in stock prices—coupled with the immaturity of China’s capital market itself, market sentiment is susceptible to influence, investors are prone to overreaction, and the risk of individual stocks quickly spread throughout the market, leading to a sharp rise and fall in the entire market phenomenon.

The abnormal stock market volatility in 2015 was the superimposed effect of such highly leveraged funding and the financial market’s herding effect. In the first half of 2015, driven by multiple favourable factors such as the central bank’s interest rate cut and lowering of quotas and the speeding up of IPO approval, the stock market rose and surged under the boost of financing, financing business, and over-the-counter funding. The network structure of the first half of the year not only increased the financial risk but also made the stock market more tightly structured. With the increase in the proportion of financing leverage, the risk of blowout increased simultaneously, after which the liquidity turned from high to low due to the rapid strengthening of financial supervision in the short term and the malicious shorting by some investors in spite of the market risk, the systemic risk increased steeply, the stock price bubble was punctured, and there were many times when a thousand shares fell, which further intensified the forced closing of positions in field financing and over-the-counter matching. This caused the stock price to cycle down, which eventually led to a dramatic fluctuation of the stock index falling by almost half within six months. With the reform and continuous improvement of China’s financial market, the market capitalisation of individual investors on the SSE dropped from 63% to 55% between 2015 and 2018, so the microstructure of the market has been differentiated in various aspects such as sectors, but overall, the institutionalisation of China’s A-share market needs to be improved in terms of both market capitalisation and trading volume.

## 6. Conclusions

Based on the daily closing prices of SSE A-shares from 2005 to 2018, this paper utilises the minimum entropy method and topological properties of networks to investigate the evolution of the SSE A-shares market from macro- and microperspectives. The main research conclusions are listed as follows:

First, stocks in the SSE A-shares market are closely connected over all periods, and the connected effect is more significant in bear markets. As a result, the degree of declines in bear markets is much greater than that of rises in bull markets. No apparent risk sources exist in the rapidly falling bear markets, but those sources can be identified in the slowly falling bear markets, which is beneficial to control.

Second, the SSE A-shares network shows the industry differentiation in the last four stages. In the first four stages, most stocks belong to the same module, referred to as the system module, which implies that risk contagion mainly appears in this module and risks transmission from the central module to peripheral modules. As the Chinese financial market develops, the growing industry aggregation in the SSE A-shares market gives rise to module differentiation and gradually undermines the system module’s status. Consequently, risks are more likely to spread in modules with similar industry categories.

Third, some stocks have consistent influences on the SSE A-shares market over eight periods, and most of them belong to health care, consumer discretionary, industrials, and materials. Status analyses suggest that a few stocks have leading effects on others and play irreplaceable roles in the network. Further, medium- and small-cap stocks with poor financial conditions are more likely to become risk sources in the SSE A-shares network, especially in the bear stage.

The Chinese financial system’s development increases investors’ risk awareness, the number of institutional investors, fundamental analysis abilities, and industry policies’ sensitivity. As a result, the SSE A-shares market is expected to increase systematic differentiation and industry aggregation. The SSE A-shares market’s long-term study reveals dynamic changes in the capital market’s microstructure, discovers the evolution of emerging financial markets, and further discusses targeted supervision on high-risk stocks. Not only can our work help investors improve asset allocations and portfolio strategies, but it can also give advice to policy makers from the view of improving market supervision and reducing market speculation.

## Figures and Tables

**Table 1:** Period division from 2005 to 2018 for the Shanghai Stock Exchange (SSE) A-shares market according to bull and bear markets.

Eight Stages	Code	Start Time	End Time	Reasons
Stage 1	Bull 1	Jun. 2005	Oct. 2007	Reforms in the market and other capital dividends.
Stage 2	Bear 1	Oct. 2007	Oct. 2008	The subprime mortgage crisis and other external factors.
Stage 3	Bull 2	Oct. 2008	Jul. 2009	Rescue policies from the government.
Stage 4	Bear 1	Jul. 2009	Mar. 2014	Combined influences from the crisis and stimulus policies
				result in market ups and downs.
Stage 5	Bull 3	Mar. 2014	Jun. 2015	Deepen reforms of state-owned enterprises and arising
				financial leverages increase the market.
Stage 6	Bear 3	Jun. 2015	Jan. 2016	Deleveraging and other factors cause the collapse of
				the stock market.
Stage 7	Bull 4	Jan. 2016	Jan. 2018	The slight rise in the market due to factors like stable leverage
				and financial stimulus.
Stage 8	Bear 4	Jan. 2018	Dec. 2018	Overseas factors like the trade war leads to the fluctuating
				decline in the stock market.

**Table 2:** Industry categories of stocks in top six modules during Bear 1.

Module Name	M1	M2	M3	M4	M5	M6
Materials	102	6	0	6	1	0
Telecommunication service	2	0	0	0	0	0
Real estate	27	6	21	3	1	0
Industrials	146	7	7	4	1	0
Utilities	38	0	1	2	0	0
Finance	13	1	2	0	0	4
Consumer staples	107	12	1	1	0	0
Energy	20	1	1	1	1	0
Consumer discretionary	54	2	0	0	0	0
Information technology	45	4	0	2	0	0
Health care	55	5	0	0	0	0

**Table 3:** Industry categories of stocks in the top nine modules during Bull 4.

Module Name	M1	M2	M3	M4	M5	M6	M7	M8	M9
Materials	2	1	8	2	1	3	2	25	1
Real estate	1	0	1	1	4	1	2	0	29
Industrials	1	2	5	21	14	7	7	2	1
Utilities	2	1	8	6	4	1	0	0	4
Finance	0	0	1	6	0	2	1	0	0
Consumer staples	5	4	2	1	1	3	11	5	3
Energy	0	0	0	0	0	0	0	0	0
Consumer discretionary	0	14	4	1	1	2	2	4	0
Information technology	1	3	1	0	2	10	0	0	0
Health care	28	8	0	1	2	1	2	0	0

**Table 4:** Industry categories of stocks in top nine modules during Bear 4.

Module Name	M1	M2	M3	M4	M5	M6	M7	M8	M9
Materials	15	3	1	1	1	18	5	0	1
Telecommunication service	0	0	0	1	0	0	0	0	0
Real estate	9	0	1	2	1	5	9	0	2
Industrials	22	4	19	16	4	3	7	4	7
Utilities	8	0	0	0	1	0	6	1	0
Finance	1	0	0	0	0	4	0	1	0
Consumer staples	20	10	3	1	18	2	1	10	0
Energy	3	0	0	0	1	8	0	0	0
Consumer discretionary	3	15	1	0	5	0	1	0	2
Information technology	7	0	27	1	2	1	1	1	0

**Table 5:** Node and link numbers of the top five modules with the highest proportion of information in eight periods.

Bull 1			Bear 1		
**Module Name**	**Node Numbers**	**Link Numbers**	**Module Name**	**Node Numbers**	**Link Numbers**
M1	488	9637	M1	608	10,887
M2	86	688	M2	44	307
M3	61	468	M3	33	224
M4	26	161	M4	19	49
M5	11	33	M5	4	8
**Bull 2**			**Bear 2**		
**Module Name**	**Node Numbers**	**Link Numbers**	**Module Name**	**Node Numbers**	**Link Numbers**
M1	528	11,755	M1	612	14,331
M2	47	329	M2	52	802
M3	51	797	M3	15	50
M4	43	533	M4	8	18
M5	20	166	M5	10	23
**Bull 3**			**Bear 3**		
**Module Name**	**Node Numbers**	**Link Numbers**	**Module Name**	**Node Numbers**	**Link Numbers**
M1	53	231	M1	306	3750
M2	47	264	M2	119	1147
M3	31	119	M3	70	500
M4	27	119	M4	72	503
M5	27	123	M5	69	504
**Bull 4**			**Bear 4**		
**Module Name**	**Node Numbers**	**Link Numbers**	**Module Name**	**Node Numbers**	**Link Numbers**
M1	40	256	M1	95	668
M2	33	211	M2	68	476
M3	30	122	M3	52	328
M4	39	201	M4	22	61
M5	29	108	M5	36	126

**Table 6:** Information flows within and between modules over eight periods.

Bull 1				Bear 1			
**Module** **Name**	**Within** **Modules**	**Flow in** **Modules**	**Flow out** **Modules**	**Module** **Name**	**Within** **Modules**	**Flow in** **Modules**	**Flow out** **Modules**
M1	66.13%	8.58%	10.82%	M1	85.87%	5.29%	5.94%
M2	13.32%	6.00%	5.41%	M2	6.80%	3.14%	2.75%
M3	10.91%	3.38%	2.29%	M3	4.65%	2.60%	2.47%
M4	3.57%	1.99%	1.87%	M4	1.89%	1.22%	1.10%
M5	2.03%	1.32%	1.14%	M5	0.58%	0.45%	0.41%
**Bull 2**				**Bear 2**			
**Module** **Name**	**Within** **Modules**	**Flow in** **Modules**	**Flow out** **Modules**	**Module** **Name**	**Within** **Modules**	**Flow in** **Modules**	**Flow out** **Modules**
M1	71.35%	9.17%	10.60%	M1	82.16%	6.24%	7.36%
M2	11.49%	4.95%	3.57%	M2	6.92%	2.90%	3.11%
M3	6.82%	2.85%	3.08%	M3	3.91%	2.40%	1.83%
M4	5.04%	2.43%	2.56%	M4	2.13%	1.40%	1.14%
M5	1.92%	1.11%	1.23%	M5	2.01%	1.29%	1.05%
**Bull 3**				**Bear 3**			
**Module** **Name**	**Within** **Modules**	**Flow in** **Modules**	**Flow out** **Modules**	**Module** **Name**	**Within** **Modules**	**Flow in** **Modules**	**Flow out** **Modules**
M1	8.34%	5.05%	5.08%	M1	46.54%	11.75%	11.49%
M2	6.58%	3.81%	3.95%	M2	18.76%	8.26%	8.20%
M3	4.59%	3.18%	3.30%	M3	10.13%	5.21%	5.08%
M4	4.41%	2.85%	2.85%	M4	8.70%	4.27%	4.31%
M5	3.15%	1.87%	1.98%	M5	7.09%	3.17%	3.43%
**Bull 4**				**Bear 4**			
**Module** **Name**	**Within** **Modules**	**Flow in** **Modules**	**Flow out** **Modules**	**Module** **Name**	**Within** **Modules**	**Flow in** **Modules**	**Flow out** **Modules**
M1	7.45%	4.30%	4.43%	M1	13.18%	7.55%	8.91%
M2	6.46%	2.60%	2.17%	M2	12.64%	4.35%	3.85%
M3	5.49%	3.72%	3.71%	M3	7.57%	3.72%	3.83%
M4	4.47%	2.45%	2.63%	M4	4.91%	3.02%	2.58%
M5	4.28%	2.71%	2.77%	M5	4.53%	2.21%	2.05%

**Table 7:** Characteristics of the SSE A-shares networks during the first four stages.

Topological Properties	Bull 1	Bear 1	Bull 2	Bear 1
Network diameter	7	6	6	7
Network density	0.0195	0.0252	0.02	0.0342
The average shortest path length	2.72	2.7	2.85	2.59
Clustering coefficient	0.071	0.078	0.055	0.097
Mean of relative degree centrality	0.02	0.0252	0.02	0.0342
Mean of relative betweenness centrality	0.23	0.234	0.255	0.22
Mean of relative closeness centrality	0.3815	0.3909	0.3699	0.4138
Out-degree centralisation	0.11404	0.05463	0.04857	0.11844
In-degree centralisation	0.03841	0.13446	0.11719	0.06242
Betweenness centralisation	0.012	0.0167	0.0308	0.0162
Out-degree closeness centralisation	0.2976	0.1937	0.1891	0.275
In-degree closeness centralisation	0.1742	0.345	0.336	0.1831

**Table 8:** Characteristics of the SSE A-shares networks during the last four stages.

Topological Properties	Bull 3	Bear 3	Bull 4	Bear 4
Network diameter	8	6	6	8
Network density	0.0165	0.022	0.0193	0.0157
The average shortest path length	3.1	2.84	3.08	3.23
Clustering coefficient	0.066	0.076	0.084	0.075
Mean of relative degree centrality	0.0165	0.022	0.0193	0.0157
Mean of relative betweenness centrality	0.28	0.255	0.278	0.296
Mean of relative closeness centrality	0.3326	0.3748	0.3389	0.3209
Out-degree centralisation	0.09127	0.01859	0.15156	0.07114
In-degree centralisation	0.04645	0.09201	0.06052	0.05434
Betweenness centralisation	0.0361	0.0172	0.0448	0.025
Out-degree closeness centralisation	0.2952	0.083	0.4127	0.2957
In-degree closeness centralisation	0.2062	0.2378	0.2306	0.2169

**Table 9:** Stocks with top five relative degree centralities in eight periods.

Bull 1		Bear 1	
**Stock code**	**Industry category**	**Stock code**	**Industry category**
600624	Health care	600340	Real estate
600218	Industrials	600085	Health care
600373	Consumer discretionary	600088	Consumer discretionary
600172	Materials	600006	Consumer discretionary
600626	Consumer discretionary	600590	Industrials
**Bull 2**		**Bear 2**	
**Stock code**	**Industry category**	**Stock code**	**Industry category**
600405	Industrials	600370	Consumer discretionary
600967	Industrials	600853	Industrials
600665	Real estate	600522	Information technology
600692	Real estate	600590	Industrials
600601	Industrials	600360	Information technology
**Bull 3**		**Bear 3**	
**Stock code**	**Industry category**	**Stock code**	**Industry category**
600460	Information technology	600410	Information technology
600439	Consumer staples	600502	Industrials
660360	Information technology	600271	Information technology
600131	Utilities	600749	Consumer discretionary
600345	Information technology	600531	Materials
**Bull 4**		**Bear 4**	
**Stock code**	**Industry category**	**Stock code**	**Industry category**
600229	Consumer discretionary	600168	Utilities
600561	Industrials	600331	Materials
600356	Materials	600292	Industrials
600757	Consumer discretionary	600713	Health care
600422	Health care	600269	Industrials

**Table 10:** Stocks with top five relative betweenness centralities in eight periods.

Bull 1		Bear 1	
**Stock code**	**Industry category**	**Stock code**	**Industry category**
600373	Consumer discretionary	600798	Industrials
600353	Information technology	600088	Consumer discretionary
600624	Health care	600811	Consumer staples
600138	Consumer discretionary	600736	Real estate
600426	Materials	600565	Real estate
**Bull 2**		**Bear 2**	
**Stock code**	**Industry category**	**Stock code**	**Industry category**
600692	Real estate	600480	Consumer discretionary
600967	Industrials	600131	Utilities
600665	Real estate	600370	Consumer discretionary
600229	Consumer discretionary	600585	Materials
600662	Industrials	600004	Industrials
**Bull 3**		**Bear 3**	
**Stock code**	**Industry category**	**Stock code**	**Industry category**
600131	Utilities	600719	Utilities
600439	Consumer staples	600410	Information technology
600460	Information technology	600533	Real estate
600166	Consumer discretionary	600502	Industrials
600879	Industrials	600501	Industrials
**Bull 4**		**Bear 4**	
**Stock code**	**Industry category**	**Stock code**	**Industry category**
600135	Materials	600331	Materials
600561	Industrials	600594	Health care
600422	Health care	600713	Health care
600757	Consumer discretionary	600375	Industrials
600730	Consumer discretionary	600390	Finance

**Table 11:** Stocks with top five relative closeness centralities in eight periods.

Bull 1		Bear 1	
**Stock code**	**Industry category**	**Stock code**	**Industry category**
600624	Health care	600085	Health care
600172	Materials	600790	Real estate
600983	Consumer discretionary	600006	Consumer discretionary
600373	Consumer discretionary	600811	Consumer staples
600985	Energy	600590	Industrials
**Bull 2**		**Bear 2**	
**Stock code**	**Industry category**	**Stock code**	**Industry category**
600405	Industrials	600370	Consumer discretionary
600967	Industrials	600522	Information technology
600692	Real estate	600853	Industrials
600665	Real estate	600480	Consumer discretionary
600787	Industrials	600861	Consumer staples
**Bull 3**		**Bear 3**	
**Stock code**	**Industry category**	**Stock code**	**Industry category**
600439	Consumer staples	600410	Information technology
600460	Information technology	600749	Consumer discretionary
600131	Utilities	600502	Industrials
600345	Information technology	600271	Information technology
600166	Consumer discretionary	600661	Consumer discretionary
**Bull 4**		**Bear 4**	
**Stock code**	**Industry category**	**Stock code**	**Industry category**
600229	Consumer discretionary	600331	Materials
600561	Industrials	600168	Utilities
600218	Industrials	600713	Health care
600757	Consumer discretionary	600292	Industrials
600422	Health care	600320	Industrials

**Table 12:** Stocks with top five PageRanks in eight periods.

Bull 1		Bear 1	
**Stock code**	**Industry category**	**Stock code**	**Industry category**
600984	Industrials	600340	Financials
600426	Materials 1	600088	Consumer discretionary
600312	Industrials	600739	Industrials
600517	Industrials	600085	Health care
600313	Industrials	600681	Utilities
**Stock code**	**Industry category**	**Stock code**	**Industry category**
600405	Industrials	600733	Consumer discretionary
600967	Industrials	600083	Consumer discretionary
600665	Financials	600585	Materials
600208	Financials	600860	Industrials
600692	Financials	600175	Energy
**Bull 3**		**Bear 3**	
**Stock code**	**Industry category**	**Stock code**	**Industry category**
600879	Industrials	600410	Information technology
600458	Materials	600749	Consumer discretionary
600131	Information technology	600502	Industrials
600746	Materials	600271	Information technology
600439	Consumer staples	600490	Materials
**Bull 4**		**Bear 4**	
**Stock code**	**Industry category**	**Stock code**	**Industry category**
600135	Consumer discretionary	600961	Materials
600843	Industrials	600351	Health care
600056	Health care	600438	Consumer staples
600439	Consumer staples	600233	Industrials
600571	Information technology	600594	Health care

**Table 13:** Stocks with top five CheiRanks in eight periods.

Bull 1		Bear 1	
**Stock code**	**Industry category**	**Stock code**	**Industry category**
600624	Health care	600006	Consumer discretionary
600218	Industrials	600126	Materials
600626	Industrials	600418	Consumer discretionary
600567	Materials	600866	Consumer staples
600237	Information technology	600976	Health Care
**Bull 2**		**Bear 2**	
**Stock code**	**Industry category**	**Stock code**	**Industry category**
600020	Industrials	600219	Materials
600601	Information technology	600808	Materials
600308	Materials	600362	Materials
600814	Consumer discretionary	600308	Materials
600360	Information technology	600853	Industrials
**Bull 3**		**Bear 3**	
**Stock code**	**Industry category**	**Stock code**	**Industry category**
600460	Information technology	600099	Consumer discretionary
600360	Information technology	600643	Financials
600345	Telecommunication services	600763	Health Care
600166	Consumer discretionary	600809	Consumer staples
600216	Health care	600167	Utilities
**Bull 4**		**Bear 4**	
**Stock code**	**Industry category**	**Stock code**	**Industry category**
600229	Consumer discretionary	600168	Utilities
600561	Industrials	600713	Health Care
600356	Materials	600292	Industrials
600218	Industrials	600269	Industrials
600327	Consumer discretionary	600353	Information technology

**Table 14:** Financial characteristics of stocks in the SSE A-shares network over eight periods. Numbers in cells and parentheses report the financial indicator averages of stocks with the top 20 centralities and all SSE A-shares.

Period	Current Ratio	Quick Ratio	Debt to Asset Ratio	Total Assets Turnover Ratio	Return on Equity	Margin Trading Balance
Bull 1	1.42 (1.38)	1.02 (1.43)	59.33 (43.03)	0.69 (0.81)	15.26 (6.05)	- (-)
Bear 1	1.42 (1.22)	0.98 (0.71)	60.09 (55.94)	0.78 (0.78)	11 (11.23)	- (-)
Bull 2	1.45 (1.55)	0.92 (0.85)	62.47 (44.29)	0.79 (0.86)	5.30 (7.40)	- (-)
Bear 1	1.71 (1.08)	1.14 (1.51)	58.95 (47.17)	0.79 (0.60)	10.18 (3.30)	1.25 (0.48)
Bull 3	1.65 (1.81)	1.26 (1.03)	54.93 (43.48)	0.77 (0.57)	10.86 (5.98)	5.14 (2.31)
Bear 3	1.94 (1.83)	1.48 (1.41)	49.28 (55.78)	0.71 (0.54)	−0.07 (−4.09)	6.52 (6.64)
Bull 4	1.95 (2.54)	1.48 (2.16)	49.84 (43.20)	0.64 (0.71)	6.82 (4.57)	4.76 (4.00)
Bear 4	1.95 (1.66)	1.50 (1.28)	50.13 (48.31)	0.66 (0.76)	−11.39 (−3.98)	4.34 (3.08)

## Abbreviations

The following abbreviations are used in this manuscript:

SSE	Shanghai Stock Exchange
RQFII	Renminbi Qualified Foreign Institutional Investor
QFII	Qualified Foreign Institutional Investor
QDII	Qualified Domestic Institutional Investor
LASSO	Least Absolute Shrinkage and Selection Operator

## Appendix B

For the LASSO estimate

(A1) $${\hat{\beta }}_{-i}=\underset{\beta_{-i}}{\mathrm{argmin}}\left\{\frac{1}{2T}\sum_{t=1}^{T}{\left(r_{it}-\sum_{j\neq i}r_{jt}\beta_{ij}\right)}^{2}+\lambda \sum_{j\neq i}|\beta_{ij}|\right\},$$

the cyclical coordinate descent algorithm [56] is used to solve the L1 optimisation problem. Computing along a regularisation path, Friedman et al. [56] also suggested that this algorithm is faster than competing methods, can handle large problems, and can efficiently address sparse features.

A coordinate descent step is considered to solve Equation (A1). Specifically, suppose the estimates for $\beta_{ij}\;\left(j\neq i,k\right)$ have been achieved, denoted as ${\hat{\beta }}_{-i}$, and the objection is to partially optimise with respect to $\beta_{ik}$. Compute the gradient at $\beta_{ik}={\hat{\beta }}_{ik}$, which only exists if ${\hat{\beta }}_{ik}\neq 0$. Denote $R_{\lambda }\left(\beta_{-i}\right)$ as the objection function in Equation (A1). If ${\hat{\beta }}_{ik}>0$, then

$${\left.\frac{\partial R_{\lambda }}{\partial \beta_{ik}}\right|}_{\beta_{-i}={\hat{\beta }}_{-i}}=-\frac{1}{T}\sum_{t=1}^{T}r_{kt}\left(r_{it}-\sum_{j\neq i,k}{\hat{\beta }}_{ij}r_{jt}\right)+\lambda .$$

A similar expression exists if ${\hat{\beta }}_{ik}<0$, and ${\hat{\beta }}_{ik}=0$ is treated separately. Simple calculus [57] shows that the coordinatewise update has the form

$${\hat{\beta }}_{ik}=S\left(\frac{1}{T}\sum_{t=1}^{T}r_{kt}\left(r_{it}-{\hat{r}}_{it}^{\left(k\right)}\right),\lambda \right),$$

where ${\hat{r}}_{it}^{\left(k\right)}=\sum_{j\neq i,k}{\hat{\beta }}_{ij}r_{jt}$ is the fitted value excluding the contribution from $r_{kt}$, and S(z,γ) is the soft-threshold operator with value

$$\mathrm{sign}\left(z\right){\left(|z|-\gamma \right)}_{+}=\left\{\begin{array}{cc} z-\gamma & \mathrm{if}\;z>0\;\mathrm{and}\;\gamma <|z|;\\ z+\gamma & \mathrm{if}\;z<0\;\mathrm{and}\;\gamma <|z|;\\ 0 & \mathrm{if}\;\gamma \geq |z|. \end{array}\right.$$

Next, iteratively update the rest estimates and obtain the final estimates ${\hat{\beta }}_{-i}$.
